# Familial Dilated Cardiomyopathy Associated With a Novel Combination of Compound Heterozygous *TNNC1* Variants

**DOI:** 10.3389/fphys.2019.01612

**Published:** 2020-01-22

**Authors:** Maicon Landim-Vieira, Jamie R. Johnston, Weizhen Ji, Emily K. Mis, Joshua Tijerino, Michele Spencer-Manzon, Lauren Jeffries, E. Kevin Hall, David Panisello-Manterola, Mustafa K. Khokha, Engin Deniz, P. Bryant Chase, Saquib A. Lakhani, Jose Renato Pinto

**Affiliations:** ^1^Department of Biomedical Sciences, College of Medicine, Florida State University, Tallahassee, FL, United States; ^2^Pediatric Genomics Discovery Program, Department of Pediatrics, Yale School of Medicine, Yale University, New Haven, CT, United States; ^3^Department of Genetics, Yale School of Medicine, Yale University, New Haven, CT, United States; ^4^Department of Pediatrics, Yale School of Medicine, Yale University, New Haven, CT, United States; ^5^Department of Biological Science, Florida State University, Tallahassee, FL, United States

**Keywords:** genetic analysis, cardiac troponin C, missense variant, dilated cardiomyopathy, *TNNC1*

## Abstract

Familial dilated cardiomyopathy (DCM), clinically characterized by enlargement and dysfunction of one or both ventricles of the heart, can be caused by variants in sarcomeric genes including *TNNC1* (encoding cardiac troponin C, cTnC). Here, we report the case of two siblings with severe, early onset DCM who were found to have compound heterozygous variants in *TNNC1*: p.Asp145Glu (D145E) and p.Asp132Asn (D132N), which were inherited from the parents. We began our investigation with CRISPR/Cas9 knockout of *TNNC1* in *Xenopus tropicalis*, which resulted in a cardiac phenotype in tadpoles consistent with DCM. Despite multiple maneuvers, we were unable to rescue the tadpole hearts with either human cTnC wild-type or patient variants to investigate the cardiomyopathy phenotype *in vivo*. We therefore utilized porcine permeabilized cardiac muscle preparations (CMPs) reconstituted with either wild-type or patient variant forms of cTnC to examine effects of the patient variants on contractile function. Incorporation of 50% WT/50% D145E into CMPs increased Ca^2+^ sensitivity of isometric force, consistent with prior studies. In contrast, incorporation of 50% WT/50% D132N, which had not been previously reported, decreased Ca^2+^ sensitivity of isometric force. CMPs reconstituted 50–50% with both variants mirrored WT in regard to myofilament Ca^2+^ responsiveness. Sinusoidal stiffness (SS) (0.2% peak-to-peak) and the kinetics of tension redevelopment (*k*_TR_) at saturating Ca^2+^ were similar to WT for all preparations. Modeling of Ca^2+^-dependence of *k*_TR_ support the observation from Ca^2+^ responsiveness of steady-state isometric force, that the effects on each mutant (50% WT/50% mutant) were greater than the combination of the two mutants (50% D132N/50% D145E). Further studies are needed to ascertain the mechanism(s) of these variants.

## Introduction

Pediatric cardiomyopathies, or diseases of the heart muscle in young patients, have an annual incidence of 1.1–1.5 per 100,000 and are the most common indication for heart transplantation in children (Lipshultz et al., 2003; Dipchand et al., 2013; Lee et al., 2017). Dilated cardiomyopathy (DCM), characterized by dilation and dysfunction of one or both ventricles, is the most common type, accounting for more than 50% of all pediatric cardiomyopathies (Lipshultz et al., 2003). Genetic variation in any of a number of proteins that affect cardiomyocyte function is an important cause of DCM (Hershberger et al., 2013; van der Velden and Stienen, 2019). Some of these variants appear to simply predispose toward cardiomyopathy, while others are a primary cause of cardiomyopathy (Burke et al., 2016).

Troponin plays a central role in contractile regulation in striated muscle. This protein complex is comprised of three distinct subunits: troponin I (TnI), troponin T (TnT), and troponin C (TnC) (Farah and Reinach, 1995). Cardiac Troponin C (cTnC), which is encoded by the *TNNC1* gene and is abundantly expressed in cardiomyocytes, functions as a myofilament Ca^2+^ sensor and plays a critical role in regulating contraction (Li and Hwang, 2015). The tertiary structure of cTnC is dominated by the two globular halves of the molecule, the C- and N-domains, which are connected by a flexible linker (Takeda et al., 2003; Li and Hwang, 2015). The C-domain binds divalent cations (Ca^2+^ and/or Mg^2+^) at two EF-hand motifs – referred to as sites III and IV based on their location in the primary sequence – and has important structural and modulatory roles in the sarcomere (Holroyde et al., 1980; Marques et al., 2017; Veltri et al., 2017a). The N-domain contains an evolutionarily defunct site I and regulatory EF-hand site II that normally triggers contraction upon Ca^2+^ binding during systole (Holroyde et al., 1980).

Missense variants in cTnC have been associated with both dilated and hypertrophic cardiomyopathies (Mogensen et al., 2004; Landstrom et al., 2008; Willott et al., 2010; Pinto et al., 2011b; Parvatiyar et al., 2012), and knockout of cTnC in adult zebrafish results in a phenotype consistent with DCM (Ho et al., 2009). Here, we report the case of two siblings diagnosed with severe, early onset pediatric DCM associated with compound heterozygous variants in *TNNC1*. As a result of our attempts to understand the molecular basis of these pediatric DCM cases, we provide *in vivo* experimental evidence confirming that *TNNC1* is a crucial gene for heart function, and further provide *in vitro* experimental evidence that altered contractile mechanics – unexpectedly – cannot always explain the pathogenicity of *TNNC1* variants.

## Experimental Procedures

### Ethics

The human subjects research in this study was approved by the Institutional Review Board of Yale University School of Medicine. *Xenopus* were maintained and cared for in an aquatics facility in accordance with Yale University Institutional Animal Care and Use Committee protocols.

### Sequencing Methods

Genomic DNA was isolated from either venous blood or saliva samples. Whole exome sequencing was performed by using IDT xGen capture kit followed by Illumina DNA sequencing (HiSeq 4000). Paired end sequence reads were converted to FASTQ format and were aligned to the reference human genome (hg19). SNVs and indels were called using a GATK pipeline and annotated using AnnoVar.

### Genome Editing in *Xenopus tropicalis*

CRISPR/Cas9-mediated genome editing in *Xenopus tropicalis* tadpoles was used as previously described (Bhattacharya et al., 2015). Briefly, two non-overlapping, independent CRISPR sgRNAs targeting *tnnc1* were designed to generate knockdowns.

CRISPR 1 oligo (targets exon 4):

CTAGCtaatacgactcactataGGTTCTTGGTCATGATGGTCgttt tagagctagaaTAGCAAG

and CRISPR 2 oligo (targets exon 5):

CTAGCtaatacgactcactataGGAGGAACTCATGCGAGATGgtt ttagagctagaaTAGCAAG.

The sgRNAs were synthesized using the EnGen sgRNA synthesis kit (NEB), and underwent subsequent purification and concentration using the RNA Clean & Concentrator-5 kit (Zymo). Individual sgRNAs were injected at 400 pg/embryo with 1.6 ng of Cas9 protein into single celled embryos according to standard methods (Ran et al., 2013). Uninjected control and CRISPR tadpoles were raised in 10 cm dishes until stage 42 to stage 45 of development. To visualize beating hearts, these tadpoles were embedded in low melt agarose in 1/9 X MR and images were obtained using a Thorlabs Telesto 1325 nm spectral domain optical coherence tomography system as previously described (Deniz et al., 2018).

### Cloning, Expression and Purification of Recombinant Proteins

Wild-type (WT) and mutant (either D132N or D145E) human cTnC proteins were cloned, expressed, and purified as previously described (Landstrom et al., 2008; Dweck et al., 2010). Site-directed mutagenesis by PCR was used to engineer point mutations into the pET3-d vector. Sequences were verified prior to expression and purification of the cTnC mutants.

### Calcium Solutions

Different Ca^2+^ solutions ranging from pCa 8.0 (relaxing) to 4.0 (activating) were calculated utilizing a pCa Calculator (Dweck et al., 2005). All solutions contained: 20 mM 3-[N-morpholino]propanesulfonic acid (MOPS), 7 mM ethylene glycol-bis(2-aminoethylether)-N,N,N′,N′-tetraacetic acid (EGTA), 15 mM phosphocreatine, 15 units mL^–1^ creatine phosphokinase, 2.5 mM MgATP^2–^, 1 mM free Mg^2+^, ionic strength maintained constant at 150 mM by adding Kpropionate (KPr), varying [Ca^2+^], pH 7.0. Anion of choice for the pCa solutions was propionate (e.g., CaPr_2_, MgPr_2_, and KPr). Solutions were prepared at room temperature (20–21°C).

### Skinned Cardiac Muscle Preparation

Left ventricular papillary muscles were harvested from porcine hearts obtained from a local abattoir and dissected into small muscle bundles (Landstrom et al., 2008). Dissected cardiac tissue was exposed to pCa 8.0 relaxation solution (10^–8^ M free [Ca^2+^]) containing non-ionic detergent Triton X-100 (1% v:v). After incubating for 4 h at 4°C, skinned cardiac muscle preparations (CMPs) were transferred to 51% glycerol-relaxing pCa 8.0 solution (v:v), stored at −20°C, and utilized within 1 month. Strips of muscle, 0.8–1.0 mm in length and 0.15–0.25 mm in diameter, were mounted using aluminum foil T-clips to a force transducer on one end and a motor on the other end. CMPs were immersed in pCa 8.0 relaxation solution and the initial length (L_0_) was set at 2.1 μm sarcomere length measured by HeNe laser diffraction. Initial maximal Ca^2+^-activated tension was measured at pCa 4.0 (10^–4^ M free [Ca^2+^]).

### Extraction of Native cTnC From CMPs and Reconstitution With Wild-Type or Mutant cTnCs

Native (endogenous) cTnC was extracted by incubating CMPs in 5 mM CDTA solution (pH 8.4) for ∼1.5 h at 21°C as previously described (Landstrom et al., 2008). To evaluate the efficacy of cTnC extraction, residual Ca^2+^-activated tension was measured by immersing the CMPs in pCa 4. CMPs exhibiting residual force >40% (n = 2) were excluded from analysis. CMPs with residual force ranging from 8.4 to 39.4% were reconstituted by incubation for ∼5 min total (five incubations of ∼1 min each) at 21°C with one of four conditions: drops of pCa 8.0 solution containing 1.0 mg mL^–1^ of either 100% WT cTnC, 50% WT/50% D132N cTnC mix, 50% WT/50% D145E cTnC mix or 50% D132N/50% D145E cTnC mix. Tension recovery was evaluated by comparing the reconstituted maximal tension at pCa 4.0 to the initial maximal Ca^2+^-activated tension measured prior to cTnC extraction (P/P_0_) and ranged from 81% to 123%.

### Muscle Mechanics

pCa-Force: Ca^2+^-dependence of steady-state, isometric force was measured by incubating reconstituted CMPs in a series of Ca^2+^ solutions ranging from pCa 8.0 to 4.0 at 21°C. Normalized [Ca^2+^]-force data for each CMP were fit using non-linear least squares regression to a 2-parameter Hill equation to obtain parameter estimates for pCa_50_ and *n*_Hill_, as described (Veltri et al., 2017a).

Kinetics of Tension Redevelopment (*k*_TR_): After force reached steady-state in each pCa solution, measurement of the rate of tension redevelopment was obtained by shortening CMPs by 20% L_0_, followed by rapid, 25% re-stretch, then release back to L_0_ (Gonzalez-Martinez et al., 2018). The apparent rate constant (*k*_TR_) was obtained from each tension recovery time course as described previously (Chase et al., 1994; Regnier et al., 1999). Conditions where steady-state, isometric force was below 15% of the maximal force were excluded from the *k*_TR_ analysis because of the relatively low signal-to-noise at the lowest levels of isometric force. Estimations of the 3-state model parameters (*f*, *g*, and *k*_OFF_) were computed in MatLab as previously described (Gonzalez-Martinez et al., 2018) with the exceptions that force for each condition was assumed to be 1.0, and also because there are no direct measurements of Ca^2+^ binding to or dissocation from the mutant cTnCs, the value for *k*_*ON*_ for all simulations was assumed to be that derived from measurements on WT, 1.84 × 10^8^ (Pinto et al., 2011a). In addition to estimates for parameters *f*, *g*, and *k*_OFF_, the modeling also provides an estimate for pCa_50_; the modified least-squares criterion is weighted such that the best fit, predicted pCa_50_ is essentially the same (well within experimental error) as that measured experimentally for the same condition.

#### Sinusoidal Stiffness

Sinusoidal stiffness (SS) was obtained by oscillating CMP length ∼0.2% L_0_ peak-to-peak at 100 Hz, and recording the length and force signals at a sampling rate of 1 kHz. SS measurements were performed and the data were analyzed as described previously (Gonzalez-Martinez et al., 2018).

### Statistical Analyses

Statistical analyses including non-linear regression were performed using SigmaPlot v.12.0. Data were tested for significant statistical differences using one-way ANOVA with *post hoc* Student-Newman–Keuls test. Data are shown as mean ± S.E.

## Results

### Clinical Data

**FIGURE 1:**
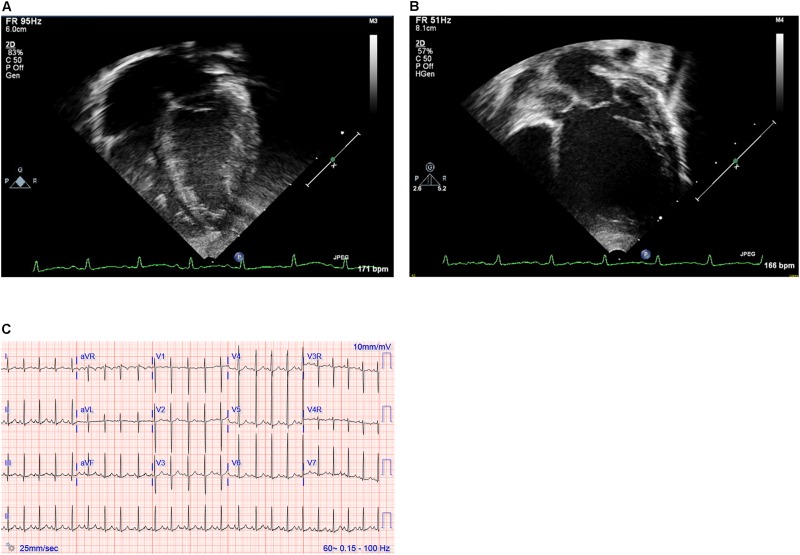
**(A,B)** Echocardiography images and **(C)** electrocardiogram. Still images obtained from echocardiogram of the proband at ages 2 weeks **(A)** and 12 months **(B)**. Note particularly the progressive dilation and prominent rounding of the left ventricle (LV) in the later image. **(C)** Electrocardiogram at 19 months of age demonstrating sinus rhythm and left ventricular enlargement.

The proband was a female delivered full term following an uncomplicated pregnancy that included two normal fetal echocardiograms. She had initially been well without symptoms referable to the cardiovascular system. She demonstrated no feeding intolerance, premature fatigue, irritability, pallor, cyanosis, increased work of breathing, tachypnea, excessive diaphoresis, or lethargy. She had a good appetite and breast fed without difficulty. Screening echocardiography was done at 18 days of age due to an older sibling with neonatal cardiomyopathy of presumed idiopathic etiology. This showed borderline enlargement of the left ventricle with a left ventricular end diastolic diameter (LVEDD) of 2.18 cm (*Z* score of 0.8) and a low decreased M-mode left ventricular ejection fraction (LVEF) of 48%. Given the family history, she was admitted to the hospital briefly with a diagnosis of DCM for initiation of medical therapy with captopril, furosemide and aspirin. She was followed closely as an outpatient, with electrocardiogram having signs of left ventricular enlargement and serial echocardiograms demonstrating gradually worsening left ventricular function, necessitating a steady escalation of her medical management (Figure 1). Notably, right ventricular size and function remained normal throughout. By 7 months of age both dilation and function of her left ventricle had worsened with echocardiography showing LVEDD 3.40 cm (Z score of 4.09) and M-mode LVEF of 32%. At 13 months of age, echocardiography showed LVEDD 4.50 cm (*Z* score of 8.55) and M-mode LVEF of 33%. She was being managed with enalapril, carvedilol, digoxin, furosemide and aspirin, and had been listed for cardiac transplantation due to her severely depressed heart function. She suffered a cardiac arrest while awaiting a suitable donor and expired at 14 months of age.

Family history was notable for an 11 year-old brother who presented in the neonatal period with poor feeding and growth, and was diagnosed with severe DCM. He was managed initially with medications then received a cardiac transplantation at 13 months of age. He has done well since, with good function of his transplanted heart, and without any signs of skeletal muscle problems. Both parents, currently in their early 40′s, have had normal echocardiograms and do not have any signs of cardiovascular disease. There is no other family history of cardiomyopathy, congenital heart defects or sudden unexpected cardiac death.

### Genetic Testing

**FIGURE 2:**
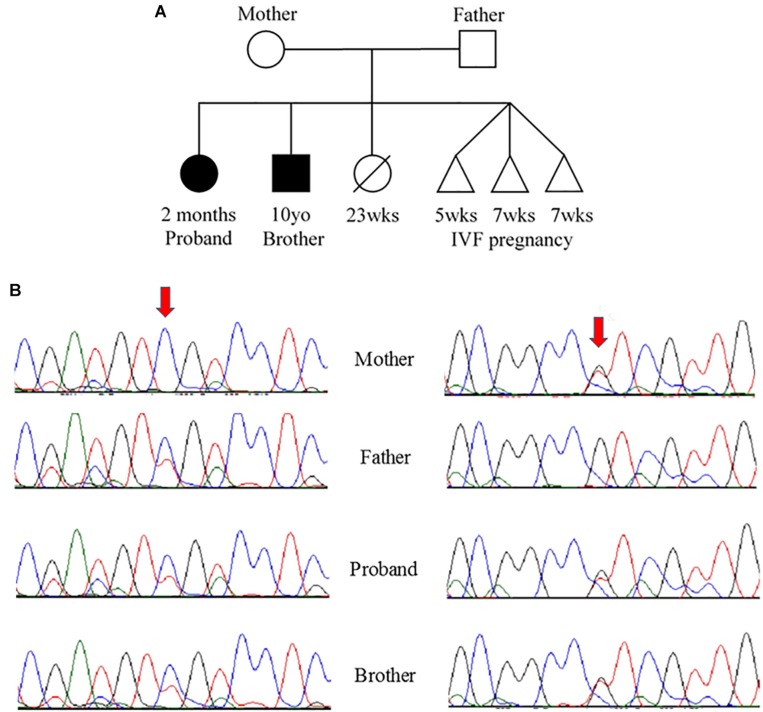
**(A)** Pedigree and **(B)** Sanger sequencing. **(A)** Family pedigree showing the proband and similarly affected brother (black shading). There was a prior loss of a severely premature live birth and three prior failed IVF pregnancies. **(B)** Sanger sequencing data from the four available family members. Red arrows indicate residue of interest. The left side shows residues around the c.394G>A change that results in D132N in one of the two alleles for *TNNC1* for three of four individuals (mother is reference, other three are heterozygous for the variant). The right side shows residues around the c.435C>A change that results in D145E in one of the two alleles for *TNNC1* for three of four individuals (father is reference, other three are heterozygous for the variant).

**FIGURE 3:**
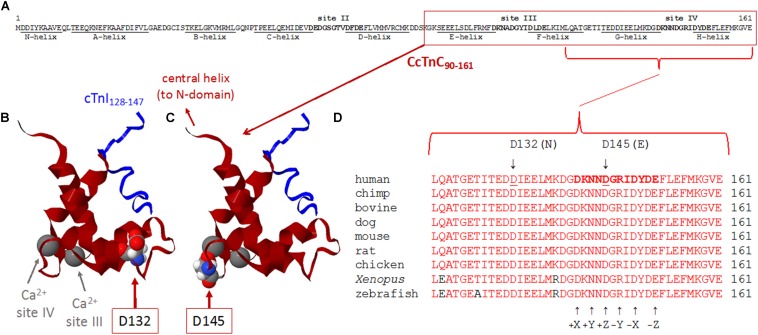
**(A–D)** Sequence and structure analyses of cTnC surrounding divalent cation-binding site IV. **(A)** Primary sequence of human cTnC (NP_003271.1). Divalent cation-coordinating regions (sites II, III, and IV) are highlighted in bold, and amino acids that comprise α-helical regions (N-helix and α-helices A–H) are underlined [annotated according to Li and Hwang (2015)]. Site II [EF-hand comprised of a-helices C–D and joining residues] is in the N-domain of cTnC and is the regulatory “trigger” site that activates contraction when Ca^2+^ binds during the systolic Ca^2+^ transient. Sites III and IV (EF-hands comprised of α-helices E–F or G–H, respectively, along with the respective residues that join each pair of α-helices) are in the C-domain and can bind Mg^2+^ or Ca^2+^. Note that all 161 amino acids of the primary sequence are intentionally shown as a single row to illustrate the relative sizes and locations of annotated regions. **(B,C)** Ribbon structures of the Ca^2+^-saturated C-domain of human cTnC in complex with a cardiac troponin I peptide (cTnI_128__–__147_), illustrating the locations of D132 and D145 (red arrows). NMR structure adapted from PDB 1OZS model 1 (Lindhout and Sykes, 2003) using MATLAB (ver. R2018b, The MathWorks, Inc.) molviewer. cTnC_90__–__161_ (dark red) and cTnI_128__–__147_ (blue) are shown as backbone ribbons, with space-filled atoms to highlight cTnC residues D132 and D145 (CPK colors). Ca^2+^ ions at sites III and IV are large gray spheres (gray arrows); note that, physiologically, it is likely that Mg^2+^ rather than Ca^2+^ would be bound at most sites III and IV in the sarcomeres of living cardiomyocytes. Labels in either panel **(B)** or **(C)** apply to the locations of structural elements in both panels. Note that only one affected amino acid is highlighted in each of panels **(B,C)**, corresponding to the occurrence of only one of the two variants in each *TNNC1* allele of the proband’s genome (Figure 2B). **(D)** Multiple alignments of residues around site IV demonstrate a remarkable degree of conservation of amino acids D132 and D145 across a wide range of species. Conserved amino acids are shown in red. Affected residues D132 and D145 are identified by underlines (human sequence) and arrows (above). D132 is within the G-helix (refer to panel **A**) that is part of site IV EF-hand. D145 is at the +Z location within the divalent cation-coordinating region of site IV; all 12 residues of this region are highlighted in bold in the human sequence (top sequence), as in panel **(A)**, and the coordinating residues (+X, +Y, +Z, –Y, –X, and –Z) within site IV are annotated (arrows) below the aligned sequences.

Due to the strong family history, the proband underwent genetic testing with whole exome sequencing, which identified two variants of uncertain significance, D132N (c.394G>A, p.Asp132Asn) and D145E (c.435C>A, p.Asp145Glu), in the *TNNC1* gene (NM_003280.2). Subsequent analysis of the family (proband, brother and both parents) revealed that these variants were inherited in *trans* (Figures 2A,B). Both variants are located in the region of divalent cation binding site IV in the C-domain of cTnC (Figure 3). Both of these aspartate residues, as well as the surrounding amino acids, are highly conserved among vertebrates (Figure 3D). In the Genome Aggregation Database (gnomAD), D145E has a frequency of 1.28 × 10^–4^, whereas D132N has not been previously reported in a healthy population. These were also noted to be the only two rare (<0.5% minor allele frequency in the general population) single gene variants across the entire exome that were shared by both affected siblings, and thus no other rare or pathogenic variants were found in other known cardiomyopathy genes in either of the siblings. The proband was sequenced to a mean depth of 102 independent reads per targeted base and her affected brother and their unaffected parents were sequenced to a mean depth of 44–49X. Greater than 10X coverage was achieved for 98% of the proband’s exome and 94% for the rest of family members’ exomes. Greater than 20X coverage was achieved for 96% of the proband’s exome and 88% for her other family members.

### *TNNC1* in *Xenopus*

**FIGURE 4:**
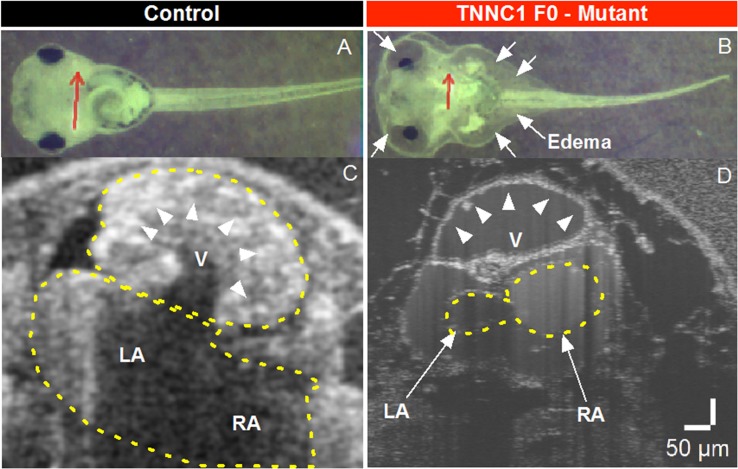
**(A,B)** Brightfield microscopy and **(C,D)** Optical Coherence Tomography (OCT) imaging of Control and *TNNC1* F0 knockout tadpoles. As compared to control tadpoles **(A)**, *TNNC1* knockout tadpoles **(B)** show normal gross morphology, but significant total body edema (white arrows) secondary to severe dilated cardiomyopathy leading to congestive heart failure. Red arrows indicate plane of OCT images in **(C,D)**. OCT images demonstrate normal size and wall thickness of cardiac chambers in control tadpole **(C)**, with severe dilation and enlargement of the ventricle in *TNNC1* knockout tadpole **(D)**, indicated with white arrowheads. V = ventricle, LA = left atrium, RA = right atrium.

To assess the consequences of altered cTnC in an *in vivo* system, we began by using CRISPR/Cas9-mediated gene editing to knockout *TNNC1* in *Xenopus*. Loss of the *TNNC1* gene did not prevent the early stages of development, and resulted in a dramatic cardiac phenotype in tadpoles consistent with DCM (Figure 4 and Supplementary Video S1). Notably, the knockout tadpoles demonstrated ventricular dilation, wall thinning and almost imperceptible cardiac motion. With the ultimate goal of introducing the two mutations separately and together, we then attempted to rescue this cardiomyopathy phenotype by expression of human cTnC in depleted frog embryos. Unpredictably, we were unable to obtain expression of cTnC in the tadpole hearts despite employing various techniques injecting either *TNNC1* mRNA or *TNNC1* plasmid DNA under control of a CMV promoter (Table 1).

**TABLE 1:** Attemps to rescue *TNNC1* knockout in *Xenopus tropicalis*.

Approach	Result
Inject human tnnc1 mRNA into fertilized embryo (one cell stage) 250 pg, 100 pg, 50 pg, 25 pg	mRNA was lethal at higher doses (≥100 pg) no other phenotype observed
Inject human tnnc1 mRNA and tnnc1 sgRNA into fertilized embryo (one cell stage) 50 pg, 25 pg	Cardiomyopathy phenotype (i.e., no rescue with reintroduction of tnnc1 mRNA)
Inject human tnnc1-GFP mRNA into fertilized embryo (one cell stage) 250 pg, 100 pg, 50 pg	See fluorescence in numerous tissues but not cardiac muscle (somites, cartilage, intestine, etc.)
Inject human tnnc1-GFP plasmid DNA (cmv promoter driven in pCSDest) into fertilized embryo (one cell stage) 100 pg, 50 pg	See mosaic fluorescence in numerous tissues but not cardiac muscle (somites, cartilage, intestine, etc.)
Inject human tnnc1-GFP mRNA into fertilized embryo (eight cell stage – D2 blastomeres to target cardiac tissue) 250 pg, 100 pg, 50 pg	See fluorescence in adjacent tissues but not cardiac muscle (intestine, liver)
Inject human tnnc1-GFP plasmid DNA (cmv promoter driven in pCSDEST) into fertilized embryo (eight cell stage – D2 blastomeres to target cardiac tissue) 100 pg, 50 pg	See mosaic fluorescence in numerous tissues but not cardiac muscle (intestine, liver)

### Muscle Mechanics

It was previously shown that cTnC-depleted CMPs reconstituted with cTnC-D145E increased Ca^2+^ sensitivity of steady-state isometric force generation (Landstrom et al., 2008; Pinto et al., 2011a; Veltri et al., 2017a). However, to our knowledge, the effects of cTnC-D132N or mixtures of these cTnCs have not been previously reported. Considering that both siblings were found to be compound heterozygous for D145E and D132N in cTnC, we sought to explore their potential functional significance *in vitro*.

Native, endogenous cTnC was extracted from porcine CMPs using CDTA incubations and reconstituted with recombinant expressed human cTnC-WT (control) or mixtures of cTnCs (section “Experimental Procedures”). The range of average of residual tension post-extraction was 18% ∼ 35%, with individual values ranging from 8.4 to 39.4%, indicating that the majority of native cTnC had been extracted from the myofilaments (Table 2, where force values are reported as specific force). Upon reconstitution, the average tension recovery was at least 87%, indicating that the majority of troponin molecules had been functionally reconstituted with exogenous cTnC and that both WT and mutant recombinant cTnCs were competent for Ca^2+^-activation of contraction.

**TABLE 2:** Hill equation parameter estimates for Ca^2+^ dependence of steady-state isometric force obtained from porcine CMPs reconstituted with exogenous cTnC WT or variants depicted in Figure 5.

	pCa_50_	ΔpCa_50_	*n*_Hill_	*F*_0__max_ $\left(\frac{\text{mN}}{{\mathrm{mm}}^{2}}\right)$	*F*_max_ $\left(\frac{\text{mN}}{{\mathrm{mm}}^{2}}\right)$	*F*_residual_ $\left(\frac{\text{mN}}{{\mathrm{mm}}^{2}}\right)$	Exp.
WT	5.43 ± 0.02	–	1.70 ± 0.20	14.87 ± 3.44	15.47 ± 3.21	2.69 ± 0.41	7
WT/D132N	5.32 ± 0.03*	−0.11	2.60 ± 0.45*	8.99 ± 1.04	11.07 ± 2.70	2.80 ± 0.60	4
WT/D145E	5.58 ± 0.04*	+0.15	1.72 ± 0.18	14.07 ± 2.05	15.77 ± 2.36	4.95 ± 0.86*	4
D132N/D145E	5.46 ± 0.02	+0.03	1.64 ± 0.14	12.32 ± 1.87	10.09 ± 1.22	2.21 ± 0.27	8

pCa_50_, pCa needed to reach 50% of the maximal force. ΔpCa_50_ = cTnC variant pCa_50_ – WT pCa_50_; *n*_*Hill*_, cooperativity of thin filament activation. Statistical significance was determined using one-way ANOVA with *post hoc* Student-Newman–Keuls test. *p < 0.05 vs. WT.

**FIGURE 5:**
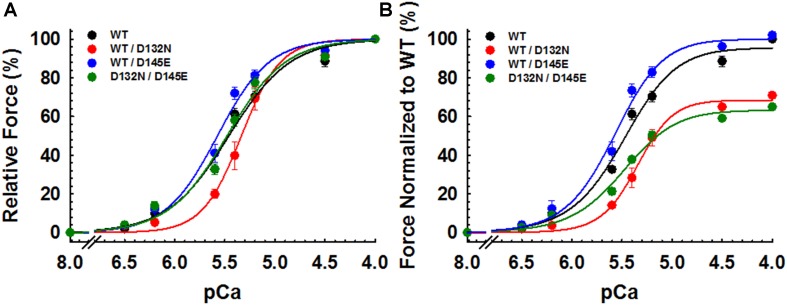
Ca^2+^ dependence of steady-state isometric tension in porcine CMPs reconstituted with exogenous cTnC WT (100% WT) or variants (50% WT/50% D132N, 50% WT/50% D145E, or 50% D132N/50% D145E). **(A)** Relative steady-state isometric force as a function of pCa. The force values were normalized to the maximal steady-state isometric force in the same preparation. **(B)** Normalized steady-state isometric force as a function of pCa. The force values were normalized to the maximal steady-state isometric force generated by WT. Data are shown as mean ± S.E. and best fit parameter estimates from non-linear least squares regression on the Hill equation are summarized in Table 2 (*n* = 4–8).

CMPs reconstituted with a mixture of 50% WT and 50% D132N variant (WT/D132N) displayed significantly reduced myofilament Ca^2+^ sensitivity of force generation (pCa_50_ = 5.32 ± 0.03), with a rightward shift of 0.11 pCa units compared with CMPs reconstituted with 100% WT cTnC (pCa_50_ = 5.43 ± 0.02) (Table 2 and Figures 5A,B). Consistent with previous reports that compared 100% D145E with 100% WT (Landstrom et al., 2008; Pinto et al., 2009; Veltri et al., 2017a), we found that reconstitution with a mixture of 50% WT and 50% D145E (WT/D145E) significantly increased myofilament Ca^2+^ sensitivity of tension (pCa_50_ = 5.58 ± 0.04), with a leftward shift of 0.15 pCa units (Table 2 and Figures 5A,B). Interestingly, we observed no significant difference in myofilament Ca^2+^ sensitivity of tension upon reconstitution of CMPs with a mixture of 50% D132N and 50% D145E (D132N/D145E) (Table 2 and Figures 5A,B). Although the D132N variant in 50%:50% combination with either WT or D145E tended to reduce maximal tension recovery, the differences were not statistically significant (Table 2 and Figure 5B). The Hill coefficient (*n*_Hill_) is an indicator of cooperativity of thin filament activation. Compared to WT (*n*_Hill_ = 1.70 ± 0.20), only the WT/D132N mixture exhibited a significant change – an increase – in the apparent cooperativity (*n*_Hill_ = 2.60 ± 0.45) (Table 2).

**FIGURE 6:**
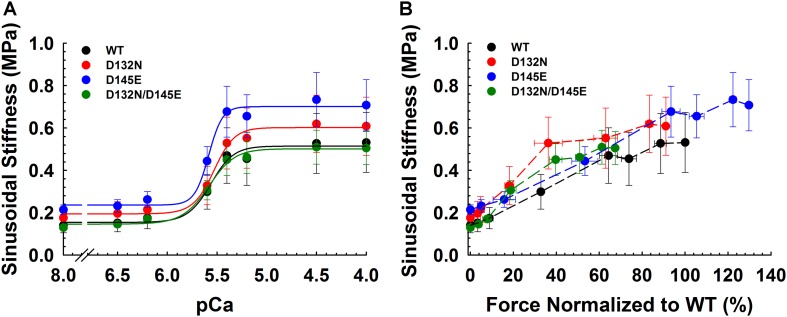
Sinusoidal stiffness analysis in porcine CMPs reconstituted with exogenous cTnC WT (100% WT) or variants (50% WT/50% D132N, 50% WT/50% D145E, or 50% D132N/50% D145E). **(A)** Ca^2+^ dependence of steady-state sinusoidal stiffness. **(B)** Normalized force vs. steady-state sinusoidal stiffness. The force values were normalized to the maximal steady-state isometric force generated by WT. **(B)** Dashed lines were drawn to connect the points. Data are shown as mean ± S.E. and maximum SS values are summarized in Table 3.

Because we observed no significant difference in myofilament Ca^2+^ sensitivity of tension for the 50%:50% combination of the two variant cTnCs (D132N/D145E) compared with 100% WT (Table 2 and Figures 5A,B), we investigated two additional indices of contractile function: SS and kinetics of tension redevelopment (*k*_TR_). SS is used as a metric of the overall number of cross-bridges, whereas *k*_TR_ is used a metric of the kinetics of cross-bridge cycling at maximal Ca^2+^-activation and also informs about thin filament regulatory unit dynamics at submaximal Ca^2+^-activation. We found no significant difference in maximum SS values when comparing CMPs reconstituted with the variants (WT/D132N; WT/D145E; D132N/D145E) to those reconstituted with 100% WT (Table 3 and Figures 6A,B), suggesting that force per cross-bridge was unchanged. In addition, there was little or no difference in any of the relationships between SS and isometric force (Figure 6B).

**TABLE 3:** Maximal sinusoidal stiffness (SS_max_) and rate of tension redevelopment (*k*_TR max_) measured in porcine CMPs reconstituted with exogenous cTnC WT or variants depicted in Figures 6, 7, respectively.

cTnC	SS_max_ (MPa)	*k*_TR max_ (s^–1^)	Exp.
WT	0.53 ± 0.14	3.24 ± 0.54	4
WT/D132N	0.61 ± 0.13	3.30 ± 0.05	4
WT/D145E	0.71 ± 0.12	3.66 ± 0.31	4
D132N/D145E	0.50 ± 0.08	2.91 ± 0.35	4

*SS_*max*_, maximum steady-state sinusoidal stiffness; *k*_*TR max*_, maximum rate of tension redevelopment.*

**FIGURE 7:**
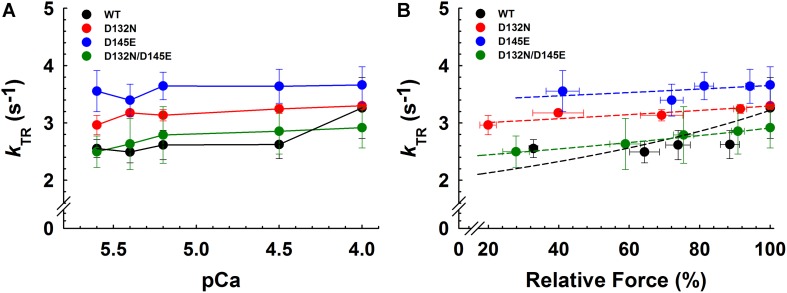
Rate of tension redevelopment (*k*_TR_) analysis in porcine CMPs reconstituted with exogenous cTnC WT (100% WT) or variants (50% WT/50% D132N, 50% WT/50% D145E, or 50% D132N/50% D145E). **(A)** *k*_TR_ as a function of increasing Ca^2+^ concentration. Solid lines were drawn to connect the points. **(B)** Relative force vs. *k*_TR_. The force values were normalized to the maximal steady-state isometric force for each preparation. Data are shown as mean ± S.E. and maximum *k*_TR_ values are summarized in Table 3. Dashed lines were obtained from fits to the 3-state model, which take into account the changes in Ca^2+^ sensitivity (Table 2 and Figures 5A,B) as described in section “Experimental Procedures”; best fit parameter estimates are summarized in Table 4.

Maximum *k*_TR_ values were not significantly different when comparing CMPs reconstituted with the variants (WT/D132N; WT/D145E; D132N/D145E) to those reconstituted with 100% WT (Table 3 and Figures 7A,B). This result informs us that, according to Brenner (1988), the sum of the sum of the rates of cross-bridge attachment and detachment (*f*+*g*) is approximately constant for all four conditions. To explore the possibility that the variants may have altered cross-bridge attachment (*f*) and detachment (*g*) rates even though the sum remained constant, we used a 3-state model of muscle regulation to evaluate the Ca^2+^-dependence of *k*_TR_ (Landesberg and Sideman, 1994; Hancock et al., 1997; Loong et al., 2013; Gonzalez-Martinez et al., 2018). The force-*k*_TR_ data for CMPs containing the mutants (WT/D132N, WT/D145E or D132N/D145E) appear to be less curvilinear than for WT (Figure 7B). The fits of the 3-state model capture the curvature of the data from CMPs containing mutants, but does not fully capture the greater curvature of the WT data (Figure 7B). Best fit values for *f*, *g*, and *k*_*OFF*_ corresponding to the lines in Figure 7B are reported in Table 4. The 3-state model’s four parameters (*f*, *g*, *k*_*ON*_, and *k*_*OFF*_) are all lumped parameters that primarily inform about cross-bridge cycling (*f* and g) and thin filament regulatory unit dynamics including Ca^2+^ binding to and dissociation from cTnC (*k*_*ON*_ and *k*_*OFF*_). Computational modeling indicates that *k*_*OFF*_ is, as expected, generally faster as Ca^2+^ sensitivity decreases (Table 4), although this relationship is modulated by additional factors because *k*_*OFF*_ reflects more than just Ca^2+^ dissociation from cTnC. Furthermore, the modeling indicates that *f* was slower than WT and *g* was faster for both WT/D132N and WT/D145E (Table 4). For the two mutants together (D132N/D145E), *f* and *g* changed in the same directions as for the individual variants (WT/D132N and WT/D145E), but the magnitudes of the changes were less; in other words, the combination of the two variants appeared to partially ameliorate the effects of the individual variants, as was also found for Ca^2+^ sensitivity of steady state isometric force (Table 2 and Figures 5A,B).

**TABLE 4:** Optimized parameter estimates and predictions from the 3-state model for force-*k*_TR_ data depicted in Figure 7.

	3-State model fitted parameters					
	Cross-bridge		Regulatory unit		3-State model predictions	
cTnC	*f* (s^–1^)	*g* (s^–1^)	*k*_*ON*_ (M^–1^ s^–1^)	*k*_*OFF*_ (s^–1^)	pCa_50_	max *k*_*TR*_ (s^–1^)
WT	1.33	1.96	1.84 × 10^8^	1123.2	5.43	3.24
WT/D132N	0.37	2.94	1.84 × 10^8^	1097.4	5.32	3.30
WT/D145E	0.30	3.36	1.84 × 10^8^	555.9	5.58	3.66
D132N/D145E	0.59	2.35	1.84 × 10^8^	856.7	5.46	2.91

*Best fit parameter estimates for three parameters (*f*, *g*, and *k*_*OFF*_) were obtained in MatLab using the Simplex method for the force-*k*_*TR*_ data in Figure 7, taking into account the Ca^2+^ sensitivity (pCa_50_) from steady-state isometric force measurements (section “Experimental Procedures”) as reported in Table 2; values for k_*ON*_ were derived from measurements reported by Pinto et al. (2011a) and maximal isometric force was assumed to be 1.0 for each dataset. These parameter sets were used to illustrate relations between *k*_*TR*_ and steady-state, isometric force in Figure 7 (dashed lines in Figure 7B).*

## Discussion

Given the central role of cTnC in cardiomyocyte contraction, it is little surprise that a growing number of variants in the *TNNC1* gene have been identified in genetic screens for familial cardiomyopathies, both hypertrophic and dilated. Here, we report two siblings with severe, early onset DCM who were found to be compound heterozygous for two *TNNC1* variants: D145E inherited from the healthy mother and D132N inherited from the healthy father. Considering that myofilament dysfunction typically correlates with severity of cardiomyopathy, we anticipated that the combination of the two variants (D132N/D145E) would have adverse consequences for Ca^2+^ regulation of contractility. However, in contrast to what might be expected from the family pedigree, our functional data indicate that these two variant cTnC proteins when present as a mixture of one variant with WT (as in the mother and father) significantly altered Ca^2+^ regulation of steady-state isometric force in opposite directions, while the combination of the two variants (as in the affected siblings) did not.

### Dissonance Between Clinical Outcomes and Mechanical Measurements

Although multiple *in silico* predictors of variant effects – including SIFT, PolyPhen2 and CADD – predict that each of these variants is likely to be detrimental, they are both classified as variants of uncertain significance in ClinVar. The D132N variant has not been reported in the Genome Aggregation Database (gnomAD). The population frequency of the D145E variant is reported as 0.0001228 in gnomAD, and the Atlas of Cardiac Genetic Variations classifies this uncommon variant as “unlikely to be pathogenic.” This classification of D145E is consistent with individuals such as the proband’s mother, who is heterozygous and asymptomatic. The available evidence, therefore, suggests that D145E could possibly be a risk factor for cardiomyopathy in some cases, but does not appear to be disease-causing when present in the heterozygous state. The two affected siblings presented here, in contrast to the parents, have D145E in *trans* with the D132N variant. When viewed in this light, the D145E population frequency is well within an expected range for a recessive disease.

To assess the combined effects of two potentially dysfunctional *TNNC1* alleles, we performed knockout experiments in *Xenopus*, revealing a dramatic DCM phenotype in tadpoles (Figure 4 and Supplementary Video S1) that is consistent with a previous report in zebrafish (Ho et al., 2009). Our multiple attempts to rescue this phenotype with human cTnC protein expression (Table 1) were unsuccessful, however, as the protein could be found expressed in other tissues but not in the heart (data not shown). The reason for this remains unclear to us at this time, though the fact that we have extensive experience obtaining wide expression – including in the heart – of multiple other proteins in tadpoles, suggests that this may be an issue specific to *TNNC1*.

Interestingly, the D145E variant was first described in a patient with hypertrophic cardiomyopathy (HCM) and it was the only variant found in a screen of fifteen HCM-susceptibility genes (Landstrom et al., 2008). In contrast, a subsequently described proband with DCM also had a rare variant in the MYBPC3 gene, which was hypothesized to combine with cTnC D145E to manifest as DCM (Hershberger et al., 2010; Pinto et al., 2011b). Based on these opposing findings, it was suggested that this second patient may have initially developed HCM that rapidly progressed to DCM (Freeman et al., 2001; Fujino et al., 2001, 2002; Nanni et al., 2003). The proband described here, however, was followed closely since birth given her brother’s diagnosis, and she never had any echocardiographic or other evidence of HCM, suggesting that this combination of cTnC alleles results purely in DCM. Of significance is another example of compound heterozygosity where the D145E variant was again associated with inherited cardiomyopathy (Ploski et al., 2016). In this case, two pediatric patients were diagnosed with autosomal recessive restrictive cardiomyopathy which was fatal during infancy. Sanger sequencing revealed that both of the affected individuals were compound heterozygous for genetic variants in *TNNC1* corresponding to D145E and A8V in the cTnC protein.

Variants in *TNNC1* can affect skeletal muscle function because cTnC is also expressed in slow skeletal muscle. The D145E mutant was reported to increase slow skeletal muscle ATPase activity at low and high calcium concentrations in reconstituted myofibrils (Veltri et al., 2017b). Although it is known that D145E does not affect myofilament calcium sensitivity in slow skeletal skinned fibers (Veltri et al., 2017b), it is unclear whether D132N has any effect on slow skeletal muscle function. Importantly, no skeletal muscle weakness was reported in the patient’s history.

### Implications for cTnC Function in the Cardiac Thin Filament

Both variants are located in the C-domain of cTnC (Figure 3). The missense D132N variant is located within the G-helix that is part of EF-hand site IV, and D145E is one of the divalent cation (Ca^2+^ or Mg^2+^) coordinating residues (+Z position) in site IV. While the regulatory site for Ca^2+^ binding is site II in the N-domain of cTnC, our prior results indicate that there is communication between the C- and N-domains (Badr et al., 2016) such that changes in the C-domain can influence the regulatory N-domain (Marques et al., 2017; Veltri et al., 2017a). The results of this study demonstrated that the D145E variant (WT/D145E) increases myofilament Ca^2+^ sensitivity even when WT is also present, while the D132N mutation (WT/D132N) had the opposite effect of decreasing Ca^2+^ sensitivity (Table 2 and Figures 5A,B) even though the two affected residues are relatively close in the primary sequence and the 3D structure (Figure 3). In general, myofilament incorporation of troponin variants that cause opposing effects on thin filament Ca^2+^ responsiveness are expected to normalize the myofilament Ca^2+^ sensitivity (Li et al., 2010; Alves et al., 2014, 2017; Dieseldorff Jones et al., 2018), and that was what was found in this study. Compared to WT, the 50%:50% mix of D132N/D145E showed similar myofilament Ca^2+^ sensitivity and Hill coefficient (*n*_Hill_) values, the latter generally reflecting cooperative processes associated with isometric force generation. While incorporation of the two mutants appeared to normalize opposite effects of the two variants on Ca^2+^ dependence of isometric force, what is difficult to reconcile is the severe DCM pathology associated with the presence of the two mutations.

A paradigm associating changes in myofilament Ca^2+^ sensitivity commonly found in HCM (increased) and DCM (decreased) is generally well-accepted (Willott et al., 2010; van der Velden and Stienen, 2019). This study, however, presents a unique clinical case in which we observed no significant changes in myofilament Ca^2+^ sensitivity due to a combination of variants in patients with DCM. How can this finding be explained? After depleting native cTnC from CMPs, each available unfilled cTnC site was presumably reconstituted, depending on the experiment, with either exogenous WT, WT/D132N mix, WT/D145E mix, or D132N/D145E mix in an attempt to mimic what is expected to be found in the patients (D132N/D145E) or the parents (WT/D132N or WT/D145E), along with the control condition (100% WT). Previous studies showed that D145E cTnC dissociates more slowly from the thin filament compared to WT cTnC indicating that D145E has a higher binding affinity for the thin filament (Marques et al., 2017), although the functional data (Table 2) do not suggest that this difference in affinity influenced the results.

As with our finding, there has been some variation in prior reports on variants in *TNNC1* with regard to the hypothesis that HCM is always associated with increased Ca^2+^ sensitivity and DCM with decreased Ca^2+^ sensitivity. The first DCM-associated variant identified in *TNNC1*, G159D (Mogensen et al., 2004), has been explored by several groups with conflicting findings. It was initially reported that G159D did not alter myofilament Ca^2+^ sensitivity in Tn-exchanged skinned rat trabeculae (Biesiadecki et al., 2007). In contrast, both cTnC-depleted skinned porcine papillary muscle and *in vitro* regulated thin filaments reconstituted with cTnC-G159D exhibited reduced myofilament Ca^2+^ sensitivity (Robinson et al., 2007; Dweck et al., 2008). Conversely, G159D exhibited increased myofilament Ca^2+^ sensitivity in both human skinned ventricular myocytes obtained from a patient bearing G159D, and thin filaments reconstituted with skeletal muscle actin, human cardiac tropomyosin, and troponin complex containing cTnC G159D (Dyer et al., 2009).

Loss of the lusitropic response (cardiac muscle relaxation) is considered a common feature of DCM and HCM (Wang et al., 2012; Dweck et al., 2014; Messer and Marston, 2014). In the case of thin filament mutants, this can be partially attributed to uncoupling of cTnI phosphorylation from modulation of Ca^2+^ sensitivity (Memo et al., 2013; Messer and Marston, 2014). Studies using TnC-extracted porcine papillary muscle fibers reconstituted with cTnC DCM-associated mutants (Y5H, M103I or I148V) exhibited diminished or abolished PKA-mediated Ca^2+^ desensitization (Pinto et al., 2011b). Although we did not test for PKA-mediated myofilament Ca^2+^ desensitization in our current study, we speculate that the presence of both mutant proteins could interfere with cTnI phosphorylation levels and functional consequences. This could cause a loss of downstream β-adrenergic stimulation which is crucial to effect the lusitropic response and diastolic ventricular filling.

Alternative possibilities exist because the two mutations are not present in the same protein. In the affected siblings, the two variant proteins are expected to be distributed randomly in thin filaments, and differences in function might affect communication between adjacent regulatory units when they contain different cTnCs. The lack of change in *n*_Hill_ for D132N/D145E (Table 2 and Figures 5A,B) suggests this might not be a plausible explanation, although the Hill coefficient represents the overall response and does not inform about highly localized effects when two variant cTnCs are present. Mechanical kinetics provides information beyond steady-state force measurement, and cooperative interactions among regulatory units do not appear to be required for fast rates of tension redevelopment, *k*_TR_ (Chase et al., 1994). Both the force-*k*_TR_ data (Figure 7B) and the 3-state model analyses of those force-*k*_TR_ relations (Table 4) suggest that the presence of either variant (WT/D132N or WT/D145E) shifts the force-*k*_TR_ relation, and alters contractile kinetics (*k*_TR_ in Figure 7B, and *f* and *g* in Table 4) in the same direction. However, both the kinetic data (Figure 7B) and modeling (Table 4) support the conclusion from analysis of the steady-state force-pCa data (Table 2 and Figures 5A,B) that the combination of the two variants, D132N/D145E, in thin filaments may partially ameliorate the effects of either mutant on contractile function. This is because D132N/D145E yields results more similar to 100% WT than either WT/D132N or WT/D145E (Table 4 and Figure 7B). Thus it appears to be necessary to seek alternative explanations, beyond changes in biomechanical function, to explain the deleterious effects of the two variants in combination in the proband and her brother.

### cTnC Function Beyond the Sarcomere

We and others have previously suggested that troponin subunits may participate in cellular functions that extend beyond contractile regulation in the sarcomere (Asumda and Chase, 2012; Wu et al., 2015; Zhang et al., 2016; Pinto et al., 2017). In fact, cTnC has been previously detected in cardiomyocyte nuclei, as well as the nuclei of certain cancer cells (Johnston et al., 2018). In addition, cTnC has been identified in the mitochondrial compartment of some cancer cell lines. Therefore, a potential mechanistic explanation for the severe disease phenotype associated with troponin mutants may be attributed to perturbations in its putative nuclear/mitochondrial function, whereby gene expression or bioenergetics could be altered by cTnC variants such as those reported here.

In summary, we report the association of compound heterozygous variants in *TNNC1* in siblings with early onset familial DCM. We provide functional evidence that both variants separately lead to abnormal myofilament Ca^2+^ sensitivity in which the variants D132N and D145E are, respectively, associated with reduced or increased myofilament Ca^2+^ sensitivity relative to WT, while these effects compensated for no change relative to WT when the two variants were combined. Analyses of activation-dependence of *k*_TR_ suggest that both variants have similar effects on contractile kinetics, although there was no significant change in maximal *k*_TR_, and that the combination of the two variants again partially ameliorated the effects of the individual variants. None of the observed changes in mechanics, however, can explain the clinical outcomes in this family. Further studies are needed to understand precisely how these variants combine to result in a severe DCM phenotype.

## Disclosure

SL is part owner of Qiyas Higher Health, a startup company unrelated to this work.

## Data Availability Statement

The raw data supporting the conclusions of this article will be made available by the authors, without undue reservation, to any qualified researcher.

## Ethics Statement

The studies involving human participants were reviewed and approved by the Institutional Review Board of Yale University School of Medicine. Written informed consent to participate in this study was provided by the participants’ legal guardian/next of kin. The animal study was reviewed and approved by the Yale University Institutional Animal Care and Use Committee.

## Author Contributions

JP and SL conceptualized the study and oversaw project administration. ML-V, JJ, WJ, EM, JT, MS-M, LJ, EH, DP-M, MK, and ED contributed to the data curation. PC contributed to computational data analysis and modeling. ML-V, JJ, JT, EM, PC, SL, and JP wrote the manuscript. All authors reviewed and approved the final version of the manuscript.

## Conflict of Interest

The authors declare that the research was conducted in the absence of any commercial or financial relationships that could be construed as a potential conflict of interest.

## Supplementary Material

VIDEO S1 Optical Coherence Tomography imaging showing contractility of tadpole hearts. Control heart (top panel) with good systolic contractility. Two views of *TNNC1*-F_0_ mutant hearts (middle and lower panels) showing minimal motion and lack of effective contractility.
